# The Apotheosis of the Tomato

**Published:** 1923-08

**Authors:** Toujours Tomates


### The Apotheosis of the Tomato.

SIR,—I am glad to see that you have been " crying up " that virtuous fruit, the tomato, which always behaves well and never does anybody any harm. I speak feelingly. I am one of those luckless mortals who have been debarred by Nature from the ability to eat raw fruit, the acids of which I find altogether maleficent. I, of course, admit that a man who can eat lobster at midnight, but cannot eat an apple, raw or baked, at mid-day, is a monstrosity ; but then many of us are monstrosities, though we may not be ready to admit it. Yet tomatoes are grateful and comforting to me at any hour, and I am now thankful for them notwithstanding that they are dear, not always ripe, and often thick-skinned. Probably they would be more widely grown if the wholesale merchants did not starve the grower and the greengrocer did not pillage the consumer.

TOUJOURS TOMATES.

